# Considering Trends in Sodium, *Trans* Fat, and Saturated Fat as Key Metrics of Cardiometobolic Risk Reduction

**DOI:** 10.5888/pcd11.140561

**Published:** 2014-12-31

**Authors:** Samuel F. Posner, Barbara A. Bowman, Janet L. Collins

**Affiliations:** Author Affiliations: Barbara A. Bowman, Janet L. Collins, National Center for Chronic Disease Prevention and Health Promotion, Centers for Disease Control and Prevention, Atlanta, Georgia.

The 2 articles by Urban and colleagues published this week in *Preventing Chronic Disease* report 15-year trends in sodium, *trans* fat, and saturated fat, 3 food components associated with increased risk for cardiovascular disease and obesity, in frequently ordered meal items (French fries, cheeseburgers, grilled chicken sandwiches, and regular cola) from leading US national fast food chain restaurants (1,2). These longitudinal findings track these 3 food components in foods that are frequently consumed by Americans. In recent surveys, almost half of Americans report eating fast food at least weekly (http://www.gallup.com/poll/163868/fast-food-major-part-diet.aspx), and similarly, nearly half report drinking soda daily (http://www.gallup.com/poll/156116/Nearly-Half-Americans-Drink-Soda-Daily.aspx). The findings by Urban et al confirm a substantial reduction in the content of *trans* fat and saturated fat in French fries but not in cheeseburgers or chicken sandwiches. Changes were inconsistent in sodium, saturated fat, and calories among food products, with the exception of sodas, where there was an increase in portion size. The authors conclude that, unlike the reduction observed in artificial *trans* fat in French fries, the content of sodium, saturated fat, and calories in the selected foods did not change much. Taken together, these findings indicate that little improvement has been made in the quality or energy density of popular fast food products and suggest the need for interventions to improve population health.

It is important to consider these findings in the larger context as public health researchers, practitioners, and policy makers develop and implement interventions to reduce intake of excessive calories, saturated fat, and artificial *trans* fat. Cheeseburgers, French fries, and a soda represent a quintessential part of American culture. Banter about them was central to the *Saturday Night Live* skit made famous by the late John Belushi. Similarly, songs made popular by performers such as Jimmy Buffett, Charlie Pride, the Gang of Four, and the Village People are all about having a cheeseburger, French fries, and a soda. These staples of the American diet are unlikely to disappear. However, central to American food choices is an unacceptably high prevalence of diet-related risk factors that compromise the health of Americans and contribute to the high costs of chronic disease. During the period examined by Urban and colleagues, the late 1990s through 2013, the US prevalence of chronic disease risk factors such as overweight, obesity, and hypertension have remained high, cardiovascular disease remains the leading cause of death, and prevalence of prediabetes and diabetes continues to increase (3). The continued popularity of fast food restaurants and continued high prevalence of diet-related risk factors remind public health researchers, practitioners, and policy makers that there is much that needs to be done.

The findings of the 2 studies by Urban and colleagues present concrete evidence on an issue that many may consider obvious: the paradigmatic meal of a cheeseburger with French fries and a soda still contains components whose consumption is associated with increased risk of adverse health outcomes. Changes in portion size can change the amount of these components consumed, as with the increasing portion size observed for regular soda, which can increase energy density.

In the past several years, increased public health efforts have sought to implement and evaluate interventions to increase access to healthy food options through farmers markets, community gardens, and other venues that make the healthier choice the easier choice whenever possible (4–6). Changes in subsidy programs and other structural changes have been implemented to remove access barriers, such as remediation of food deserts or placement of farmers markets in high-traffic areas, such as near subway stops. Many evaluation efforts have focused on measuring consumer awareness and purchasing behavior as surrogates for consumption (5–7). Few studies have evaluated changes in key outcomes, including consumption patterns, changes in risk factors, or changes in health outcomes. These 3 outcomes are challenging to study because they are hard to measure, the time horizon to document an effect is long, and the attribution to a specific intervention is difficult to make. Researchers, practitioners, and policy makers need to work together to develop creative methods to study these important outcomes. Evaluating effect and return on investment of these interventions is critical to identify which interventions will make a lasting difference in the health of the population. The experience with *trans* fat suggests that regulatory interventions, including menu labeling in restaurants and vending machines, to be implemented in 2015 (http://www.fda.gov/NewsEvents/Newsroom/PressAnnouncements/ucm423952.htm), will improve the food environment. New federal nutrition policy, including the 2015 update of the *Dietary Guidelines for Americans*, will help shape the environment to support healthier choices. At the same time, there is increased public concern about the healthfulness of fast food and growing support for efforts to increase access to healthy food options, particularly in schools. Taken together, expanded policy levers in concert with increased consumer awareness and demand for healthy food products will accelerate improvement in the US diet and food environment and support reductions in the population’s cardiometabolic risk.
